# Effect of socioeconomic status on wait times for patients undergoing treatment for laryngeal conditions in a universal healthcare system

**DOI:** 10.1002/lio2.930

**Published:** 2022-10-18

**Authors:** Katie de Champlain, Saud Sunba, Shari Beveridge, Meri Andreassen, Doug Bosch, Derrick R. Randall

**Affiliations:** ^1^ Section of Otolaryngology – Head & Neck Surgery, Department of Surgery, Cumming School of Medicine University of Calgary Calgary Alberta Canada; ^2^ Cumming School of Medicine University of Calgary Calgary Alberta Canada; ^3^ Calgary Voice Program Alberta Health Services Calgary Alberta Canada

**Keywords:** laryngology, socioeconomic status, wait times

## Abstract

**Objectives:**

As one of the world's only fully publicly administered, universal healthcare systems, Canada intends to provide equitable access to services for all patients. Socioeconomic status (SES) can affect treatment wait times with implications on health outcomes; however, this has not been evaluated in the setting of laryngeal disease, which incorporates urgent and elective conditions, in a universal healthcare system. This study aims to identify whether SES‐affected treatment wait times for laryngeal therapies in this system.

**Methods:**

A retrospective review was conducted on a cohort of patients with laryngeal disease at an academic, tertiary center who underwent laryngeal surgery over a three‐year period. Data retrieved through medical records; surgical and voice therapy wait times were normalized to each practitioner's average wait time for respective diagnostic categories. Income was used to assess SES and was derived from Statistics Canada census information based on patient postal codes.

**Results:**

Data analysis identified 578 patients (59% male). Mean wait time to surgery for all conditions was 123.5 (95% confidence interval 113.1–133.9) days. Analysis of variance analysis found no difference in wait times between different SES groups (*p* = .28), regardless of laryngeal disease category. Patients with cancer or airway obstruction had shorter wait times compared with benign conditions (*p* < .0001).

**Conclusions:**

SES did not affect treatment wait times for laryngeal therapies in a universal healthcare system. Wait times were shorter for oncologic and obstructive conditions compared with benign conditions, reflecting an ability to accommodate clinical needs without impacting care access at the detriment of different SES statuses.

**Level of Evidence:**

4

## 1 INTRODUCTION

Wait times for surgery can have implications on health outcomes and quality of life for individuals, but they depend on triaging based on diagnosis and urgency in order to provide access to a limited resource. Canada operates a universal healthcare system with the goal of ensuring equitable access to healthcare services for all patients. To ensure the healthcare system provides urgent access to patients with the greatest risk if left untreated, the province of Alberta developed the Alberta Coding Access Targets for Surgery (ACATS) to monitor and manage surgical wait list times.^1^ This is a standardized system that prioritizes surgery based on diagnosis and level of urgency, where elective surgeries are classified as non‐urgent and should be scheduled on a first seen basis. On the other hand, urgent surgeries should be scheduled into timeslots recognizing need for earlier intervention within the finite constraints of operating room and service provider capacity. Within this system, various factors may impact the duration of time from assessment to treatment, such as surgeon preference, patient priorities, or inherent bias toward elements such as socioeconomic status (SES).

SES is the social standing or class of an individual or group of individuals and is measured by a combination of education, income, and occupation.^2^ There is widespread literature on inequities that exist within the healthcare system and how patients with lower SES face barriers to care.^3^ Studies from several different nations and healthcare systems identified variable impacts of SES on access to care, perception of health, and outcomes.^4^, ^5^, ^6^, ^7^, ^8^ SES could potentially impact elective surgical wait times, and if such disparities exist within the healthcare system these need to be identified and addressed accordingly. However, there is limited evidence on how SES can impact wait times for surgical care, specifically within the Canadian healthcare system.

Laryngeal diseases represent a broad spectrum of conditions that include non‐urgent elective issues, and urgent or emergent conditions where time to intervention appropriately differs between them. Another layer of complexity unique to laryngeal issues relates to the potential negative impacts on employability, absenteeism, and general public perception that occurs among patients with voice or respiratory disorders that may impact need for intervention beyond traditional life and limb considerations to improve quality of life.^9^, ^10^, ^11^, ^12^, ^13^, ^14^, ^15^ Furthermore, certain laryngeal conditions are treated nonsurgically through voice therapy and respiratory training methods, which involves speech pathologists. This adds another parallel wait time system as those treatment options span multiple sessions rather than a single operation that attempts to treat the condition. There is a paucity of literature related to the social determinants of health in the timely treatment of laryngeal diseases, which is a significant knowledge deficit given the psychosocial impacts of voice in an individual and society.

The Calgary Voice Program offers surgical and non‐surgical interventions for patients with laryngeal pathology. Individuals that present to the clinic come from a wide range of backgrounds and the program strives to provide equitable healthcare to all patients, regardless of SES. The goal of this study was to identify if SES affects wait times for voice care within a high‐volume subspecialty practice managed in a universal healthcare system.

## 2 METHODS

### 2.1 Patient selection and study design

The study was a retrospective review of a cohort of participants who underwent treatment for laryngeal disorders between January 1, 2017, and March 1, 2020. Due to the global coronavirus pandemic that was declared in March 2020, we excluded data obtained after March 1, 2020. Eligible participants were identified through the Calgary Voice Program electronic medical records. Patients were required to be 18 years of age or older, undergoing their first laryngeal treatment and have available postal code information to meet our inclusion criteria. Laryngeal treatments included voice therapy and surgical management. Exclusion criteria included patients under the age of 18 years, prior laryngeal treatments, or primary address outside Alberta. Research ethics approval to conduct the study was obtained by the University of Calgary Conjoint Health Research Ethics Board (protocol REB20‐0761).

Patient demographics, date of procedural consent, date of procedure, and final diagnosis were extracted from electronic medical records. Area‐based SES measures were gathered using postal codes and extrapolated from 2016 Canadian census values, with average income defined by specific postal code used as a measure for SES. Income quintiles defined by Statistics Canada did not align with quintiles within the study population, so these were redefined to match our population within five evenly distributed quintiles.

### 2.2 Statistical analysis

Laryngeal diseases were organized into six categories, according to underlying pathology and depending on whether they were treated surgically or nonsurgically. Surgical conditions were grouped in airway disorders (mainly subglottic and posterior glottic stenosis), laryngeal cancer, benign midmembranous lesions of the larynx designated for surgical treatment, recurrent respiratory papillomatosis (RRP), and unilateral vocal cord paralysis. Patients with RRP were considered a separate category due to the potential for different wait times compared with cancer or benign disorders related to potential diagnostic uncertainty, airway impingement, or burden of disease. Patients planned for treatment with voice therapy included muscle tension dysphonia, vocal tremor, spasmodic dysphonia, paradoxical vocal fold motion, and phonotraumatic lesions intended for nonsurgical therapy (vocal fold nodules, pseudocysts, and nonspecific inflammatory change).

Data were analyzed using IBM SPSS Statistics (Armonk, New York) software. A one‐way analysis of variance (ANOVA) was conducted to evaluate if there were statistical differences between the average wait times for various laryngeal pathologies between the SES groups. To account for potential differences in treatment time between different surgeons and speech‐language pathologists due to practice patterns as well as to account for different ACATS treatment windows, wait times for each patient were normalized to the mean wait time for each practitioner, for each diagnosis. A *t*‐test analysis was performed to determine if differences between average wait times for individual diagnostic categories were truly significant. Chi‐squared testing analyzed the distribution of different diagnosis categories between the different SES groups. A *p‐*value < .05 was considered significant.

## 3 RESULTS

### 3.1 Patient characteristics and laryngeal diagnoses

**FIGURE 1:**
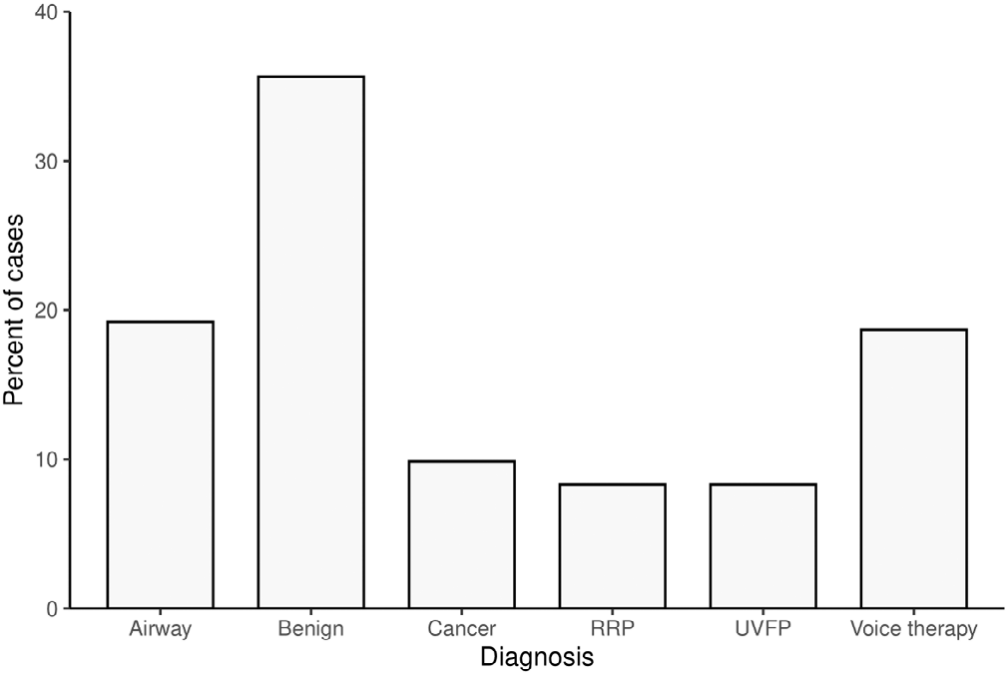
Distribution of diagnoses for all patients (*n* = 578). Benign mid‐membranous lesions constitute the largest proportion of patients, with each category containing sufficient patients for accurate assessment. RRP, recurrent respiratory papillomatosis; UVFP, unilateral vocal fold paralysis

A total of 578 patients (59% male) who presented to the Calgary Voice program between January 1, 2017, to March 1, 2020, met inclusion criteria. The mean age was 57.5 years (range 21–91). The average wait time to procedure for all patients and diagnoses was 123.5 days (95% confidence interval 113.1–133.9; Table 1). The most common diagnoses were benign midmembranous lesions (35.6%). Recurrent respiratory papillomatosis and unilateral vocal fold paralysis were the least common diagnoses (Figure 1).

**TABLE 1:** Patient demographics

Factor	Total	Female	Male
Gender (%)	—	59	41
Age (years ± SD)	57.6 ± 15.6	56.3 ± 15.3	59.4 ± 16.4
Time to treatment (days ± SD)	124 ± 127	121 ± 143	125 ± 115

### 3.2 SES and ACATS urgency effects on wait times

Income quintiles were generated for our data (Table 2). A chi squared test showed no significant difference in distribution of diagnoses between the different SES groups (*Χ* ^2^ = 15.462, *p* = .749). One‐way ANOVA identified no significant differences in normalized wait times between the different SES groups within each diagnostic category (Table 3).

**TABLE 2:** Income quintiles for study population

Quintile	Study population values (CDN$)
First (lowest)	<51,328
Second	51,328–60,930
Third	60,931–69,127
Fourth	69,128–77,637
Fifth (highest)	>77,637

**TABLE 3:** Analysis of variance evaluating standardized wait times for different clinical entities

Diagnostic group	Source	SS	df	MS	*F*	*p* value
Airway	Between groups	6.17	4	1.54	1.64	**.17**
	Within groups	99.87	106	0.94		
	Total	106.04	110			
Cancer	Between groups	5.27	4	0.32	0.77	**.55**
	Within groups	88.93	52	1.71		
	Total	94.20	56			
Benign	Between groups	7.00	4	1.75	1.79	**.13**
	Within groups	196.21	201	0.98		
	Total	203.21	205			
RRP	Between groups	1.33	4	0.33	0.65	**.63**
	Within groups	21.92	43	0.51		
	Total	23.25	47			
UVFP	Between groups	0.32	4	0.079257567	0.32	**.86**
	Within groups	10.60	43	0.246582447		
	Total	10.92	47			
Voice therapy	Between groups	14,479.37	4	3619.84127	0.5	**.74**
	Within groups	747,616.74	103	7258.41492		
	Total	762,096.10	107			

Abbreviations: df, degrees of freedom; MS, mean squares; RRP, recurrent respiratory papillomatosis; SS, sum of squares; UVFP, unilateral vocal fold paralysis.

**FIGURE 2:**
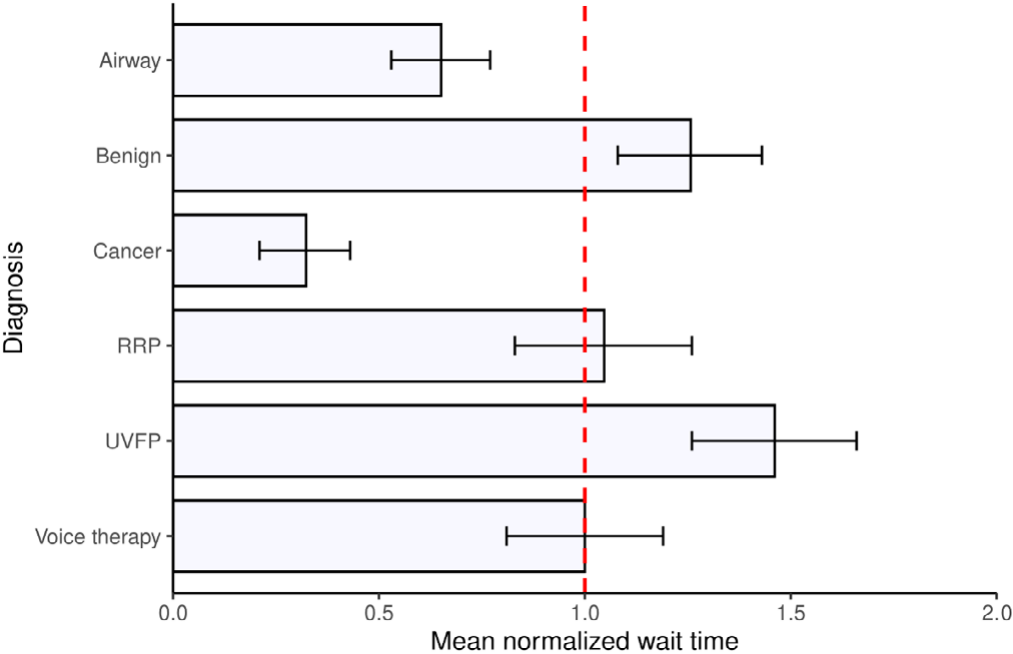
Wait times based on diagnoses normalized to surgeon and speech‐language pathologists average wait time. Error bars reflect 95% confidence intervals. The vertical bar at 1.00 represents the average wait time for all procedures. RRP, recurrent respiratory papillomatosis; UVFP, unilateral vocal fold paralysis

Cancer and airway stenosis cases are given higher ACATS priority codes and accordingly waited less than the average wait time compared with other diagnoses, as shown in Figure 2. To evaluate whether this was statistically different, the two groups were combined into an urgent case category and compared with the mean wait time for nonurgent diagnoses via a *t*‐test as a secondary analysis, which confirmed urgent cases waited statistically less time between diagnosis and treatment (*p* < .0001).

## 4 DISCUSSION

Managing patients with laryngeal disorders is a multifaceted task that purposefully accounts for acuity of disease, impacts on a patients' functioning capacity and quality of life, and numerous other factors. However, disparities in healthcare provision are commonly associated with implicit biases, and despite being unintentional, they can have significant negative effects on patients from psychosocial or physical aspects.^16^, ^17^, ^18^ Identifying factors that impact healthcare delivery is critical to shaping healthcare reform and promoting equitable access. This is increasingly important in resource‐limited settings, and surgery is a classic paradigm given finite operating room time. This is the first study to assess implications of SES on wait times in a surgical subspecialty within Canada, a universal healthcare system with well‐known issues related to surgical wait times, and the first study evaluating how SES impacts treatment times for patients with established laryngeal disease. The results of this study demonstrated SES does not impact treatment wait times for laryngeal surgical procedures or voice therapy, while still accommodating need for urgent access for patients with potentially life‐threatening disease.

Disparities in surgical care delivery can have profound impact on patients' outcomes. There are limited studies investigating the relationship between SES and wait times for surgery, and of those available there have been mixed results. A recent study conducted on wait times for elective general surgery in Vancouver, British Columbia, found no relationship between SES and surgical wait times; however, they did report that patients with lower SES tended to have worse overall perception of health.^5^ Another Canadian‐based study examined 22 common procedures performed in Ontario and found that only prostatectomy showed statistically significant findings: those of higher SES accessed intervention earlier.^4^ Studies conducted in other regions in the world have shown mixed results—Norwegian researchers found no effect of SES on surgical wait times while one study conducted in Australia found that higher SES was associated with shorter wait times for elective surgery.^6^, ^19^ Evidence from nine European countries shows that higher education was found to be associated with lower surgical wait times; however, income had mixed results.^7^ Our study enhances the current literature by providing information on a subspeciality population in a public healthcare system.

A strength of this study includes the large, comprehensive population, as the majority of voice and airway disorder population treated through surgery or voice therapy in our health region is managed through the surgeons and speech‐language pathologists involved in this study. This study found equitable access to resources provided through the Calgary Voice Program. Patients access the Calgary Voice program through provincial healthcare coverage which is free of cost; however, our study did not consider other factors that may contribute to inequitable healthcare. For example, medications that may be required in the preproceddure or postprocedure outpatient course are not covered by the healthcare system. However, lack of medication coverage is a common pitfall of the Canadian healthcare system and is not specific to laryngeal pathology. Patients in the lower SES groups may not have drug plan insurance through their occupation, or potentially from unemployment, and therefore may experience a higher burden of disease due to the inability to readily access these medications.

In our study, airway and cancer cases were found to have statistically shorter wait times compared with other diagnoses, aligning with Alberta's healthcare model of providing those with greatest need access first. One consideration is whether laryngeal diseases are different than other disorders since urgent disorders are potentially life‐threatening, yet benign disorders causing dysphonia impact a key aspect of personal identity, with voice being essential to how a person expresses themselves and is perceived by others and impacts interactions. Studies have shown that patients with longer surgical wait times are associated with higher disease burden and reduced quality of life.^20^, ^21^, ^22^ This poses the question for future studies to investigate whether the quality of life should be factored into surgical access planning.

Certain limitations should be taken into consideration for this study. First, this was a retrospective cohort study, and as a result, individual income data could not be obtained, so area‐based measures were used to quantify an individual's income status. While these have been shown to be a good representation of income in studies evaluating a large population, since this will narrow a patient's site of residence to within one or two city or neighborhood blocks (on average representing 10 households in urban areas or 500 households in rural regions), it could potentially affect the accuracy of the results.^8^, ^23^, ^24^, ^25^ For example, area‐based measures do not account for whether a person residing in that area owns or rents the property, so this can be a potential source of error. To provide a more robust assessment of SES other variables could be considered including occupation or educational attainment; however, we could not reliably achieve this for the entire study population as information was found to be inconsistent in electronic health records. Nonetheless, counterarguments can be made for area‐based SES measures' sufficient accuracy given the tendency for people to reside within areas that reflect similar occupations, educational status, ethnicity, gender identity, and other aspects contributing to overall SES, as well as the challenge for accounting for different income among retirees whose site of residence match income from their working lifetime rather than current income. When we reviewed the income quintiles generated for our population, we recognized they were higher on average than the Canadian population quintiles, so this may represent misclassification of some patients from lower SES categories; however, statistical analysis using the Canadian quintiles for our population did not impact our results. This infers that our sample population quintiles did not mask any true differences between SES thereby increasing the validity of the findings. An additional factor our study did not evaluate was the timeline from referral to the Calgary Voice Program and initial visit. It is possible that there may be differences in this timeline due to SES, which has been identified as a factor in previous studies within the Canadian and American healthcare systems among laryngeal cancer patients.^26^, ^27^ Previous studies have shown that patients within the lower SES quintiles and limited drug coverage will be more likely to cancel appointments.^28^ However, referral triage times are complex given the uncertainty of whether disease is present based on referral information—this study population includes patients previously seen by other otolaryngologists as well as undifferentiated patients referred from primary care providers—so this study chose to focus on whether treatment delays existed for different SES groups in the setting of established laryngeal disease or dysfunction. Future studies could consider how SES may impact these aspects of the patient care model.

## 5 CONCLUSION

This study reviewing the effect of SES on laryngeal surgical and voice therapy treatment wait times found no difference between different SES groups. Airway and cancer cases have appropriately statistically shorter average wait times compared with other procedures, implying that more urgent cases are being triaged and prioritized without detriment to members of any SES category. Overall, the information obtained from this study confirms that the universal healthcare system structure aligns with its goal of providing equitable access to care for all patients.
